# Upregulation of neuroglobin expression and changes in serum redox indices in a rat model of middle cerebral artery occlusion

**DOI:** 10.3892/mmr.2015.3593

**Published:** 2015-04-03

**Authors:** AIJIA SHANG, YING YANG, HANGYAN WANG, JING WANG, XINGYI HANG, ZHONGFENG WANG, DINGBIAO ZHOU

**Affiliations:** 1 Departments of Neurosurgery, General Hospital of Chinese People’s Liberation Army, Beijing 100853, P.R. China; 2 Health Medicine, General Hospital of Chinese People’s Liberation Army, Beijing 100853, P.R. China; 3 Pediatrics, General Hospital of Chinese People’s Liberation Army, Beijing 100853, P.R. China; 4 National Center for Scientific Data Sharing for Population and Health, Beijing 100730, P.R. China; 5 Medical Neurobiology of State Key Laboratory, Fudan University, Shanghai 200032, P.R. China

**Keywords:** middle cerebral artery occlusion, neuroglobin, hypoxia-inducible factor-1α, redox index

## Abstract

Neuroglobin (NGB) is a recently identified protein, which is localized in the neurons and retinal cells of the central and peripheral nervous systems in vertebrates. It is hypothesized to function as a scavenger for reactive oxygen species, or as a stress-responsive sensor for signal transduction in hypoxic-ischemic brain insults. However, the mechanism underlying the physiological function of this protein remains to be elucidated. In the present study, the profiling of changes in the serum redox index of morphological features of the hippocampus and cortex, and of the expression of NGB and hypoxia-inducible factor-1α (HIF-1α), are described in a rat middle cerebral artery occlusion (MCAO) model. The necrotic zone of the rat neural tissues increased in size with increasing reperfusion time, and different brain slices exhibited necrosis in different regions. The number of NGB-positive hippocampal and cortical cells, as well as NGB and HIF-1α transcript and protein levels in the ischemic cortex, increased with increasing reperfusion time. NGB and HIF-1α mRNA and protein levels peaked in the group that received reperfusion at 32 h after MCAO. These findings indicated that HIF-1α may be involved in ischemic pathology in an MCAO model and that NGB expression may be upregulated. Serum superoxide dismutase (SOD) activity decreased and serum malondialdehyde (MDA) levels increased with increasing reperfusion time, indicating that the redox potential increased following MCAO. Serum SOD and MDA measurements may, therefore, be useful as biomarkers for the early detection of ischemic injury in a clinical setting.

## Introduction

Neuroglobin (NGB) was identified and initially described by Burmester *et al* in 2000 (1). This recently identified protein is expressed in the tissues of the nervous system, including those of the retina (2). It is a member of the hemoglobin superfamily and is a significant ischemic-hypoxic biomarker for brain injury (3–5). An increase in the expression of NGB under hypoxic conditions exhibits a neuroprotective function *in vitro* and *in vivo* (6–8). The oxygen-binding properties of NGB are comparable to those of typical vertebrate myoglobin, suggesting a similar function for NGB in the brain (9–11). Previously, NGB upregulation in the murine brain following forebrain ischemia, has been demonstrated following carotid artery occlusion (12). However, compared with extensive brain ischemia, focal cerebral ischemia, such as basal nucleus infarction, is more frequently observed in clinical settings (13).

Consequently, the determination of whether NGB is upregulated during focal ischemia, and whether such upregulation exerts a neuroprotective effect near the ischemic penumbra, was important. Furthermore, a primary objective of the present study was to identify upstream proteins, which exhibit ischemia-induced changes in expression. In addition, the variation in serum redox index values in focal brain ischemic cases was of interest, such as those for superoxide dismutase (SOD) and malondialdehyde (MDA), as this may indicate oxygen radical-induced lipid peroxidation during hypoxic-ischemic encephalopathy, thereby providing an index of neuronal damage and recovery. The present study was designed to characterize changes in the expression of NGB and other ischemia-regulated proteins in brain tissue, as well as to profile serum redox indices in a rat model of focal cerebral ischemia following reperfusion for different time periods.

## Materials and methods

### Animals

The present study was approved by the ethics committee of the General Hospital of the Chinese People’s Liberation Army (Beijing, China). A total of 63 male Sprague-Dawley rats (weight, 280–300 g; Vital River Laboratory Animal Technology Co. Ltd., Beijing, China) were randomly divided into the following seven groups (each containing nine rats): The sham group, in which the common carotid artery (CCA) was exposed, without insertion of a filament; the 0 h reperfusion group, in which middle cerebral artery occlusion (MCAO) was performed but no reperfusion treatment was administered; and five ischemic-reperfusion groups, in which MCAO was performed and reperfusion treatment was administered for 4, 8, 16, 32 or 64 h, after MCAO treatment. All experimental procedures and animal handling protocols were approved by the Institutional Animal Care and Use Committee of the General Hospital of the Chinese People’s Liberation Army (approval no. 2008-X1-71). The animals were allowed *ad libitum* access to food and water and housed in a climate-controlled environment (25°C).

### Construction of rat MCAO model

The MCAO model was established according to the following procedures. Rats were anesthetized with 1% sodium pentobarbital (40 mg/kg; Sigma-Aldrich, St. Louis, MO, USA). A midline neck incision was made in order to expose the right CCA and to enable its separation from the adjacent nerves and tissues. The internal carotid artery was isolated, following occlusion of the external carotid artery, and a filament was inserted into the CCA by scalp acupuncture and slowly advanced until resistance was felt, while a portion of the filament remained exposed. The filament was removed following 1.5 h of mechanical artery blockage. Sham surgery was performed in an identical manner, but without filament occlusion of the arteries. The animals in the six groups in which MCAO was performed were sacrificed using 1% sodium pentobarbital (80 kg/kg) following reperfusion, 0, 4, 8, 16, 32 or 64 h after MCAO treatment.

### Triphenyltetrazolium chloride (TTC) staining

Rat brain tissues were frozen at −20°C for 20 min and cut into 10-*µ*m sections using a cryostat (LS-3000; Shenyang Longshou Electronic Equipment Co., Ltd, Shenyang, China). The sections were labeled P1, P2, P3, P4 and P5, and were stained with 2% TTC (Sigma-Aldrich) at 37°C for 20 min in complete darkness. The necrotic areas were analyzed using Image-Pro Plus 7.0 software (Media Cybernetics, Inc., Rockville, MD, USA).

### Hematoxylin and eosin (HE) staining

The rat brain tissues were prepared, fixed and sliced prior to storage. HE staining was performed according to the following procedure. Preserved slides were deparaffinized and rehydrated prior to staining. Frozen or vibratome sections were mounted on slides and rehydrated prior to staining. A slight over-staining of the sections with HE (Sigma-Aldrich) was performed for 3–5 min, depending on the section thickness and quantity of fixative present. Excessive stain was then removed using tap water. The differentiation was accomplished with four-five immersions in acidic alcohol or until sections appeared red. Excess alcohol was removed by rinsing with tap water. The nuclei were stained blue by treating the HE-stained sections with bicarbonate for 2 min, followed by an 8 min rinse with tap water. The HE-stained sections were placed in 70% ethanol for 3 min and then stained with eosin for 2 min in order to resolve the cellular details. Eosin-treated sections were subjected to three consecutive treatments of 5-min incubations with 95% ethanol followed by transfer to absolute ethanol for clearing. Images of stained hippocampus and cortex sections were captured through a microscope (80i; Nikon, Tokyo, Japan) connected via a charge-coupled device camera (magnification, x200; Nikon Corporation, Tokyo, Japan).

### Immunohistochemistry

Sections were deparaffinized, rehydrated and washed three times with 0.01 M phosphate-buffered saline (PBS). Endogenous peroxidase activity was quenched by incubating the sections with 3% H_2_O_2_ (Beijing Zhongshan Biotechnology Co., Ltd., Beijing, China) for 30 min. The sections were then subjected to sequential incubations in 10% normal goat serum (Beijing Zhongshan Biotechnology Co., Ltd.) in 0.01 M PBS for 30 min at room temperature. The sections were incubated in polyclonal rabbit anti-goat NGB antibody (1:100; cat. no. sc-22001; Santa Cruz Biotechnology, Inc., Santa Cruz, CA, USA) in PBS, containing 0.3% Triton X-100 at 4°C overnight. Following three washes for 5 min each with PBS, the sections were incubated in peroxidase-conjugated goat anti-rabbit IgG (cat. no. 111213-96-8; 1:200; Zymed, San Francisco, CA, USA) for 1 h at room temperature. Finally, the sections were developed with diaminobenzidine (Sigma-Aldrich) in 0.1 M Tris-buffered saline, containing 0.001% H_2_O_2_ for 30–50 min. The number of NGB-positive cells and total positive area in the assigned sub-regions was measured using the Image-Pro Plus 7.0 software.

### Reverse transcription quantitative polymerase chain reaction (RT-qPCR) for hypoxia inducible factor (HIF)-1α and NGB

The PCR primer pairs used, which were based on HIF-1α, NGB, and β-actin sequences from rats, were as follows: Forward: 5′-GATGCAGCACGATCTCGGCGAA-3′ and reverse: 5′-TGGGAGCTCACGTTGTGGGGAA-3′ for HIF-1α, forward: 5′-AAGGGCGGTTCTCTGGGAGCTT-3′ and reverse: 5′-AGAGGATGTGCAGGGCCAGCTT-3′ for NGB and forward: 5′-GATGCAGCACGATCTCGGCGAA-3′ and reverse: 5′-TGGGAGCTCACGTTGTGGGGAA-3′ for β-actin.

Rat brain tissues from each group were separated into ischemic and non-ischemic regions. Total RNA was extracted from tissues using TRIzol™ reagent (Invitrogen Life Technologies, Carlsbad, CA, USA), and reverse transcribed using the Total RNA transcription kit (cat. no. R6834-01; Omega Bio-Tek, Inc., Shiga, Japan). For RT-qPCR, 100 ng total RNA was used as a template quantity in order to determine HIF-1α and NGB mRNA expression levels. The results were analyzed using SDS 1.4 software (Applied Biosystems, Foster City, CA, USA), based on the 2^(−ΔΔCt)^ method (14).

### Western blot analysis of NGB and HIF-1α expression

The total brain tissue protein for each group was extracted and quantified. Approximately 35 mg total protein was separated by 12.5% SDS-PAGE and transferred onto a polyvinylidene difluoride membrane for overnight hybridization with polyclonal rabbit anti-rat NGB antibody (1:500; sc-22001; Santa Cruz Biotechnology, Inc.), polyclonal rabbit anti-mouse β-actin (1:1,000; sc-81178; Santa Cruz Biotechnology, Inc.) and monoclonal rabbit anti-rat HIF-1α antibody (1:500; ab51608; Abcam, Cambridge, MA, USA). The blotted membranes were incubated for 2.5 h with horseradish peroxidase-labeled goat anti-rabbit secondary antibody (1:1,000; cat. no. sc-2004; Santa Cruz Biotechnology, Inc.). The protein bands were read with an electronic scanner and analyzed using the Image-Pro Plus 7.0 software.

### Quantification of serum SOD activity and MDA concentration

The SOD enzyme activity and MDA concentration were measured according to the manufacturer’s instructions using SOD and MDA assay kits (Dojindo Laboratories, Beijing, China).

### Statistical analysis

All data are expressed as the mean ± standard deviation. The statistical analysis for the morphometric quantification of the NGB-positive cells was performed using a one-way analysis of variance. Scheffé’s test for group mean comparisons was used for post-hoc comparisons. Statistical analyses were performed using SPSS version 21.0 software (IBM Corp., Armonk, NY, USA). P<0.05 was considered to indicate a statistically significant difference.

## Results

### Necrotic zone areas increase with increasing reperfusion time and different brain slices exhibit different necrotic zones

**Figure 1:**
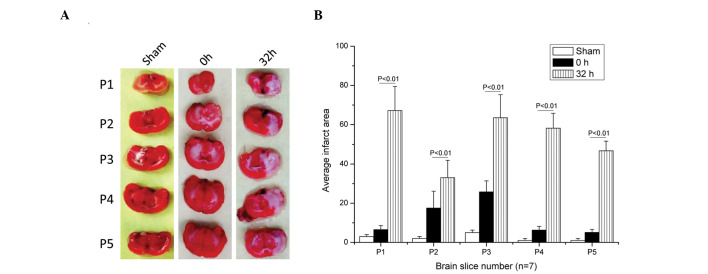
Necrotic zone assay of rat brain tissue sections stained with TTC. (A) Brain sections stained with TTC from the sham, 0 h reperfusion and 32 h reperfusion groups. (B) Histogram for comparison of the necrotic zones in different brain sections, indicating that necrotic zone areas increased with increasing reperfusion time and different brain slices exhibited different necrotic zone (P<0.01). TTC, Triphenyltetrazolium chloride.

Different brain sections exhibited different necrotic zones, as indicated by TTC staining. The ischemic-reperfusion group exhibited larger necrotic zones than that of the 0 h reperfusion and sham groups in each brain section (P<0.01; Fig. 1A). The reperfusion treatment groups exhibited significant damage in different brain sections, as compared with the sham and 0 h reperfusion groups (P<0.01; Fig. 1B).

### Quantity of hyperchromatic cells in different hippocampal and cortical regions increases with increasing reperfusion time

**Figure 2:**
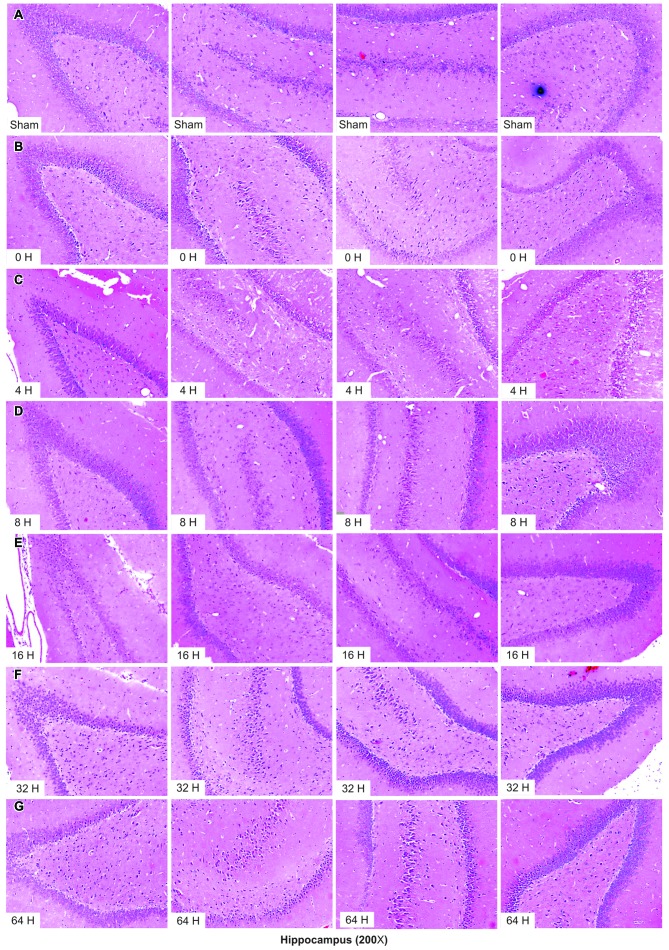
Morphological structure assay of the hippocampus using hematoxylin and eosin staining. (A)–(G) Morphological structure changes in the hippocampus in the sham group and following different reperfusion time periods (0, 4, 8, 16, 32 and 64 h; magnification, x200).

**Figure 3:**
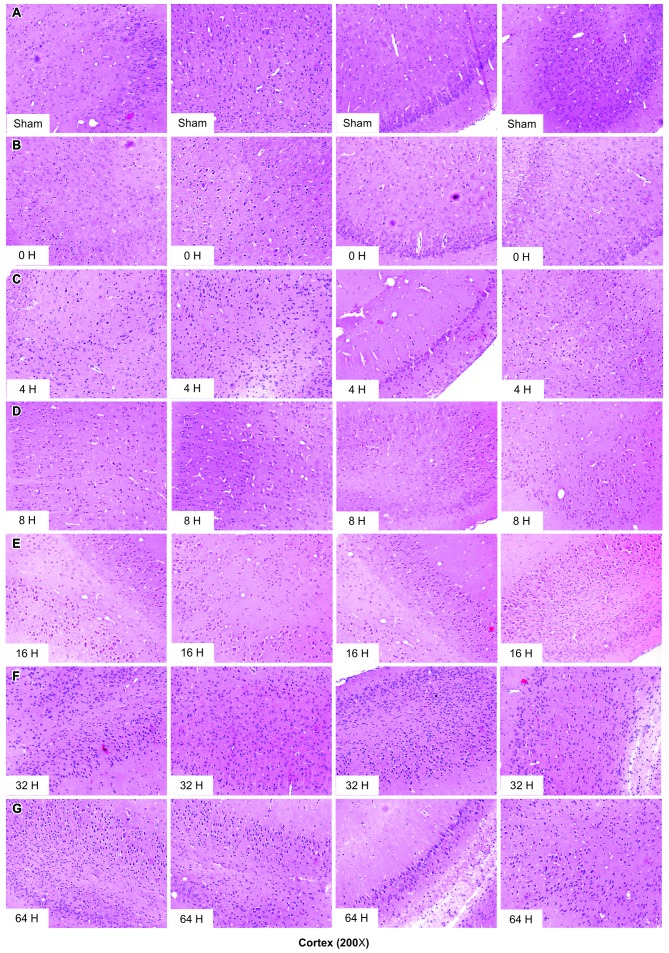
Morphological structure assay of the cortex using hematoxylin and eosin staining. (A)–(G) Morphological structure changes in the cortex in the sham group and following different reperfusion time periods (0, 4, 8, 16, 32 and 64 h; magnification, x200).

The number of hyperchromatic cells in different hippocampal regions detected by HE staining, increased with reperfusion time and peaked in the group that received reperfusion at 32 h after MCAO (Fig. 2). A similar trend was observed for cortical regions (Fig. 3).

### Quantity of NGB-positive cells in different cortical regions increases with increasing reperfusion time

**Figure 4:**
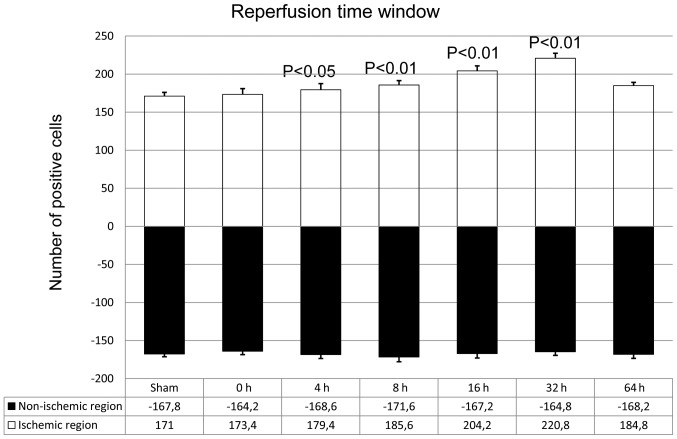
Cortical neuroglobin expression in ischemic and non-ischemic regions following induced focal cerebral ischemia, as determined by immunohistochemistry. (P<0.05 and P<0.01, compared with sham control).

Following induction of acute focal cerebral ischemia, NGB expression levels in the ischemic region increased with increasing reperfusion time, as indicated by immunohistochemical analysis. The maximal level of NGB expression was observed in the group that received reperfusion at 32 h after MCAO (P<0.01; Fig. 4). However, NGB expression levels in the non-ischemic regions did not vary with reperfusion time (P<0.01; Fig. 4).

### NGB and HIF-1α mRNA and protein expression levels increase following ischemic reperfusion

**Figure 5:**
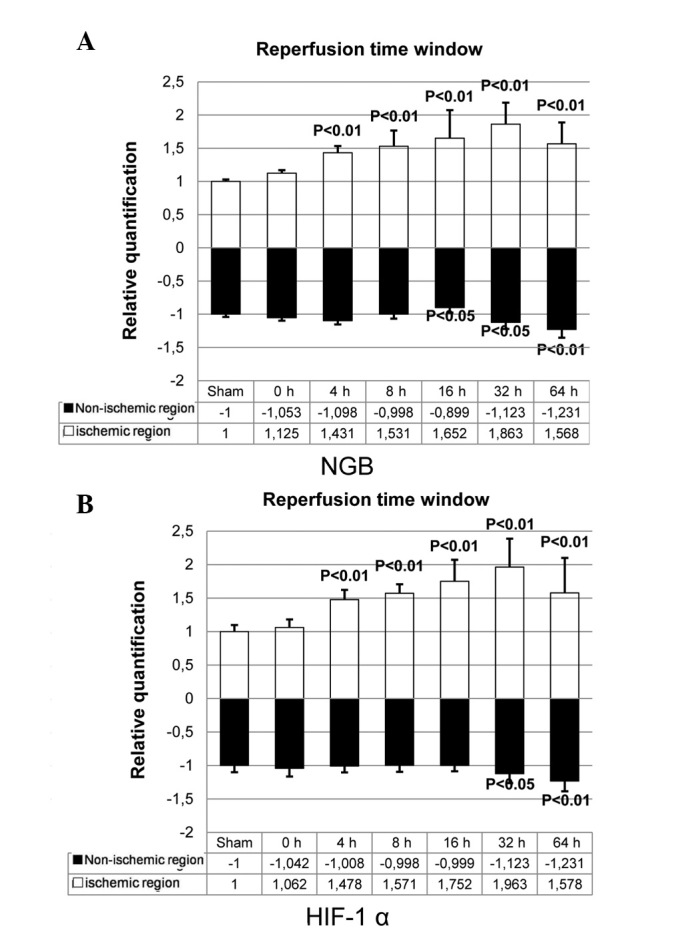
HIF-1α and NGB mRNA expression levels in ischemic and non-ischemic regions, as determined by reverse transcription-quantitative polymerase chain reaction. (A) NGB mRNA level in the treatment and sham groups. (B) HIF-1α mRNA level in the treatment and sham groups. (P<0.05 and P<0.01, compared with sham control). HIF, hypoxia inducible factor; NGB, neuroglobin.

NGB expression levels in the ischemic region increased with increasing reperfusion time; the levels for all five ischemic-reperfusion groups were significantly higher than that of the sham group, and levels peaked in the group that received reperfusion at 32 h after MCAO (P<0.01; Fig. 5A). For the non-ischemic regions, NGB expression levels in the 16 h and 32 h reperfusion groups were reduced, compared with those in the sham group (Fig. 5A), and the decrease was significant in the group that received reperfusion at 64 h after MCAO (P<0.01; Fig. 5A). It was also observed that the HIF-1α expression level increased in the ischemic region with increasing reperfusion time, and was significantly higher in all five ischemic-reperfusion groups than that in the sham group. The HIF-1α expression levels peaked in the group that received reperfusion at 32 h after MCAO (P<0.01; Fig. 5B). By contrast, in the non-ischemic regions, the mRNA transcription level of HIF-1α was unchanged at 16 h of reperfusion, although it increased in the group that received reperfusion at 32 h and 64 h after MCAO (P<0.05, P<0.01; Fig. 5B).

### NGB protein levels in the ischemic regions markedly increase with increasing reperfusion time and are significantly higher for all ischemic-reperfusion groups compared with the sham group

**Figure 6:**
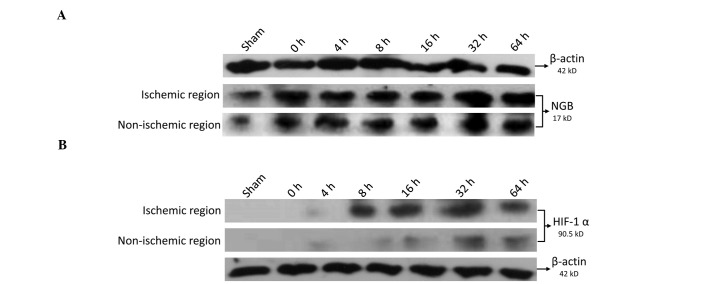
HIF-1α and NGB protein expression levels in ischemic and non-ischemic regions, as determined by western blot analysis. (A) NGB protein expression level in the treatment and sham groups. (B) HIF-1α protein expression level in the treatment and sham groups. HIF, hypoxia inducible factor; NGB, neuroglobin.

The protein levels of NGB peaked in the group that received reperfusion at 32 h after MCAO (Fig. 6A). The NGB protein level in the non-ischemic region also increased with increasing reperfusion time and was significantly higher for all ischemic-reperfusion groups compared with the sham group, while it was lower than that in the ischemic region (Fig. 6A). Regarding HIF-1α protein level in the ischemic regions, low levels of expression were observed after 4 h reperfusion, and the expression level markedly increased from 8 h of reperfusion until 64 h reperfusion, with maximal levels observed in the group that received reperfusion at 32 h after MCAO (Fig. 6B). However, HIF-1α protein expression was markedly lower in the non-ischemic regions (Fig. 6B).

### Changes in SOD activity and MDA levels in the serum following focal cerebral ischemia

SOD activity decreased with increasing reperfusion time and rapidly increased in the group that received reperfusion at 64 h after MCAO. (P<0.05, P<0.01; Table I). By contrast, serum MDA concentrations increased with increasing reperfusion time and decreased after 64 h reperfusion (P<0.05, P<0.01; Table I).

## Discussion

In the present study, the necrotic zone produced by cerebral ischemia was characterized, demonstrating an increase in the number of hyperchromatic cells and NGB-positive cells in cortical tissues. It was observed that NGB and HIF-1α mRNA and protein expression levels increased with increasing reperfusion time, with peak expression levels in the group that received reperfusion at 32 h after MCAO. The expression levels of the two proteins in the ischemic-reperfusion groups were significantly different from those in the sham group. The measurements of serum redox indices revealed that the SOD enzyme activity decreased with increased reperfusion time, although it rapidly increased in the group that received reperfusion at 32 h after MCAO. The MDA level, by contrast, increased with increasing reperfusion time and decreased in the group that received reperfusion at 32 h after MCAO.

The present results indicated that mRNA and protein levels of NGB increased in rat brain tissues between 4 h and 64 h following focal cerebral ischemia, with significant transcriptional and translational upregulation in the ischemic regions, although not in the non-ischemic regions. These findings suggested that NGB may be important in sensing and responding to focal cerebral ischemia. In order to address whether proteins other than NGB were upregulated in the MCAO model, HIF-1α levels were examined, and changes in its mRNA and protein levels were observed. These results demonstrated that HIF-1α expression patterns were similar to that of NGB. Therefore, it was hypothesized that NGB is involved in the hypoxic response to MCAO via upregulation of HIF-1α. Future studies are required to examine the regulatory interactions between NGB and HIF-1α, and more closely examine the signaling pathway governing NGB expression in the MCAO model.

Redox index assays for SOD activity and MDA concentration, revealed significant changes from 4 h of reperfusion following focal cerebral ischemia. The direction of change in SOD activity was opposite to that of MDA levels and similar to that of changes in NGB expression. Therefore, the serum SOD and MDA changes may be useful biomarkers for brain focal cerebral ischemia, as these can be easily measured (15–17). For patients with focal cerebral ischemia, the most effective way to reduce further nerve damage is to administer reperfusion treatment as soon as possible. However, even with advanced imaging techniques, such as computed tomography (18) and magnetic resonance imaging (19,20), early detection of focal cerebral ischemia is difficult. According to the present experimental results, the measurement of serum SOD activity may be a novel, accurate and convenient diagnostic approach for identifying focal cerebral ischemic pathology at an early stage.

In conclusion, NGB levels were upregulated following focal cerebral ischemia, and were shown to increase with increasing reperfusion time. The present findings provide preliminary evidence that serum SOD activity and MDA concentrations may be used as biomarkers of early focal cerebral ischemia, due to the simplicity and accuracy of their assay methods.

## Figures and Tables

**Table I:** SOD activity and MDA concentration in the serum following different periods of ischemia reperfusion.

Reperfusion time (h)	Group	
	SOD (U/ml)	MDA (nmol/ml)
Sham	114.157±0.967	5.177±0.123
0	97.317±1.366^a^	5.403±0.047
4	85.100±2.011^b^	5.910±0.061^a^
8	80.547±0.359^b^	6.443±0.146^b^
16	73.767±1.842^b^	6.840±0.098^b^
32	70.667±0.358^b^	7.533±0.061^b^
64	88.860±1.363^b^	6.910±0.070^b^

a P<0.05 and

b P<0.01, compared with the sham group. Data are presented as the mean ± standard deviation. SOD, superoxide dismutase; MDA, malondialdehyde.
